# Attack of the clones: An NK cell origins story

**DOI:** 10.1016/j.omto.2023.02.010

**Published:** 2023-03-14

**Authors:** Joseph R. Caporale, Dean A. Lee

**Affiliations:** 1 Abigail Wexner Research Institute of Nationwide Children’s Hospital, Columbus, OH, USA; 2 Graduate School of Biomedical Sciences, The Ohio State University, Columbus, OH, USA

Natural killer (NK) cells are extremely diverse, driven by germline genetic diversity between individuals and by phenotypic heterogeneity within individuals. This diversity is extremely important clinically, as specific phenotypes correlate to NK cell performance and directly relate to patient outcomes in a wide range of human conditions—from cancer to infectious diseases to pregnancy.^1^ While diversity in the NK cell phenotype and its association with outcomes has been well characterized, it has been difficult to understand its developmental mechanisms or maintenance *in vivo* or to act on that knowledge toward better *ex vivo* expansion methods for cellular therapy. Diversity within NK cell therapeutic products manufactured for clinical use has not been well defined, and it would be helpful to understand whether clonal or phenotypic subsets were selected, restricted, or maintained during *ex vivo* expansion. Moreover, how do these *ex vivo* methods compare—with regards to NK cell diversity—with that of endogenous NK cells during homeostatic or virus-driven *in vivo* expansion? In this issue, Allan et al. leverage a unique animal resource to isolate NK cell phenotypes and track clonal repertoire during *ex vivo* expansion^2^ and find that clonal contributions are maintained and clonal phenotypes have some degree of plasticity.

Investigating these questions has been problematic for the NK cell field, as well—established methods in T and B cell research are not applicable to NK cells. In particular, NK cells do not possess the highly diverse genetically rearranged receptors (T cell receptor [TCR] or B cell receptor [BCR]) that can be tracked with fine clonal specificity for T and B cells. Moreover, despite this lack of somatic alterations to drive diversity, there is still tremendous diversity seen in NK cells. Differential expression of 30+ surface receptors is determined stochastically through epigenetic regulation and environmentally driven selection, effecting a variegated receptor expression that results in up to 30,000 distinct NK cell phenotypes in a healthy individual.^3^ In previous work by these authors,^4^ these problems were addressed by implementing genetic barcoding at the hematopoietic stem cell level in a small cohort of rhesus macaques and validating the ability to follow those cell-specific barcodes during NK cell development *in vivo* and their relationship to NK cell phenotypic diversity.

Here, analysis of the starting cell composition provided the authors with the barcode repertoire present in the mature peripheral blood NK cells of each animal. As with humans, macaques showed wide variation in their initial NK cell clonal diversity, with a 5-fold range in number of initial clones. NK cells from these monkeys were then expanded using lymphoblastoid cell line^5^ or K562 feeder cell^6^ approaches that are in common use for clinical-grade expansion of therapeutic NK cells. The authors then tracked and quantified the barcodes of the expanding NK cell cultures and compared them with starting populations to define patterns of loss or gain. Over 50% of clones present in the starting population persisted in culture during the 15-day expansion period, suggesting broad maintenance of clonality. The relative change in proportion of those clones was quite diverse, however, suggesting that some clones responded better than others to the expansion conditions.

NK cells were also sorted into subpopulations using surface receptors to better understand the clonal contribution to phenotypic diversity. In one set of experiments, the authors sorted populations based on expression of KIR3DL01 (KIR3DL^+^ or KIR3DL^−^). The KIR3DL^+^ clones showed persistence during expansion, whereas KIR3DL^−^ clones tended to decline. Separately, CD56- and CD16-expressing clones were evaluated. The authors concluded that CD56^+^ clones were biased toward maintaining CD56 expression, whether as CD56^+^CD16^−^ or CD56^+^CD16^−^ populations, while CD16^+^-biased clones maintained CD16 expression as CD56^−^CD16^+^ or CD56^+^CD16^+^ populations. Altogether, the results indicate at least some degree of phenotypic plasticity in all three receptors, although the study is somewhat limited to broader conclusions about stability or plasticity of phenotype by the lack of high-dimension characterization of diversity. This was somewhat constrained by the availability of antibodies to macaque NK cell surface markers.

**Figure 1:**
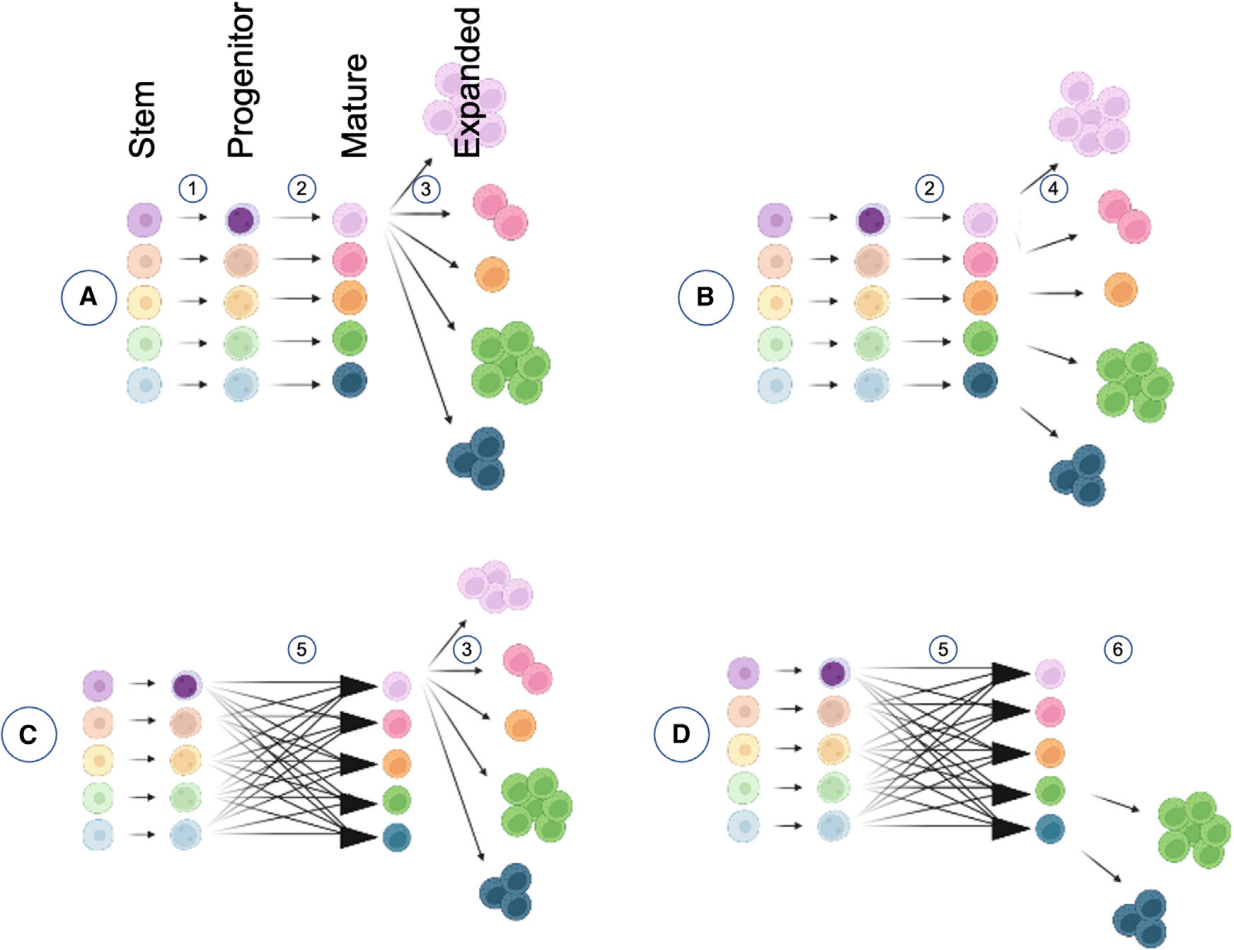
Models for differentiating clonal diversity from phenotypic diversity across NK cell development and expansion Cells are represented at key stages of differentiation and marked for representation of clonal and phenotypic diversity, showing several hypotheses for how the two might change across time. (A) Clonal and phenotypic diversity is synchronous and maintained *in vivo* (1 and 2). Expansion is clonally biased, resulting in clonal loss, but phenotypic diversity is maintained through later stage plasticity (3). (B) Clonal and phenotypic diversity is synchronous and maintained *in vivo* (1 and 2) and is fixed at early stages *in vivo* and during expansion without plasticity. (C) Clonal diversity is maintained *in vivo* but is asynchronous with phenotype because of later-stage plasticity (5). Expansion is clonally biased, resulting in clonal loss, but phenotypic diversity is maintained through continued plasticity (3). (D) Clonal diversity is maintained *in vivo* but is asynchronous with phenotype because of later-stage plasticity (5). Expansion is clonally biased, resulting in clonal loss, and loss of plasticity also constrains phenotypic diversity (6).

Although this unique set of animals has given us a rare and valuable opportunity to view the relationship between hematopoietic clonality and diversity, there remain some questions around NK cell plasticity across the developmental spectrum. In general, bland phenotypic uniformity at the stem cell level gives way to phenotypic diversity at maturity. Clonal diversity, however, may be synchronous or asynchronous with phenotypic diversity. If clonal diversity is lost, phenotypic diversity may also be lost if phenotypic potential is constrained and defined at early stages, or it may be maintained or even enhanced if there is sufficient plasticity during later stages. There are many possibilities to be considered, which are only narrow a little by these studies (Figure 1).

This work has implications in understanding the clonal origins of *de novo* NK cell diversity, NK cell reconstitution after stem cell transplantation, and diversity in the translational application of NK cell therapy and therefore raises many new questions. Are there correlations between expansion and phenotype, or are there non-phenotypic clonally dependent factors? Can further dissection of high and low-expanding clones or high and low-expanding phenotypes help us understand what drives expansion? Can these discoveries work backward into understanding *in vivo* expansion and natural variations in responses to cancer and viral infections?

This research sets the foundation for new understandings of NK cell clonal and phenotypic diversity in the setting of NK cell immunotherapy. As we continue to unravel the complexity of NK cell development and the clonal relationships to function and phenotype, manipulating clonal responses may allow for the more targeted expansion of specific isolated NK cell populations that will be more effective against certain diseases.
